# Epidemiological Investigation and Phylogenetic Analysis of Bovine Respiratory Disease Complex in Northern China

**DOI:** 10.1002/vms3.70608

**Published:** 2025-09-18

**Authors:** Yaping Zhou, Ting Guo, Hongmei Zhao, Yongqing Hao

**Affiliations:** ^1^ Laboratory of Microbiology and Immunology, School of Veterinary Medicine Inner Mongolia Agricultural University Hohhot Inner Mongolia China; ^2^ Inner Mongolia Academy of Agricultural and Animal Husbandry Sciences Hohhot Inner Mongolia China

**Keywords:** bovine respiratory disease complex (BRDC), co‐infection, northern China, pathogen diversity, phylogenetic analysis

## Abstract

Bovine respiratory disease complex (BRDC), a multifactorial syndrome driven by viral and bacterial co‐infections, poses significant challenges to cattle health in northern China. We performed a large‐scale epidemiological and phylogenetic investigation (2022–2024) to identify BRDC pathogens in the region. A total of 5052 samples from symptomatic Holstein calves (nasal swabs, sera, tissues) were analysed using virological, bacteriological and molecular methods. Viral pathogens—bovine viral diarrhoea virus (BVDV), bovine respiratory syncytial virus (BRSV), bovine parainfluenza virus‐3 (BPIV‐3) and bovine herpesvirus‐1 (BHV‐1)—dominated infections, with pronounced seasonal peaks in colder months. The prevalence of non‐cytopathic BVDV highlighted clinical difficulties. Bacterial infections involved *Mycoplasma bovis* (*M. bovis*), *Mannheimia haemolytica* and *Pasteurella multocida*, with *M. bovis* prevalent in mixed infections. Phylogenetic analysis revealed biological connections between Chinese isolates and international strains (e.g., BVDV subgenotypes 1a/1d and BPIV‐3c), showing global pathogen flow. Transmission electron microscopy revealed the presence of viral popular structure. Meanwhile, seasonality strongly influenced viral dynamics, while bacterial detection remained stable, involving environmental and management factors. Pathogen co‐infections demonstrate the complexity of BRDC. This study provides the first comprehensive BRDC pathogen profile for northern China, emphasising the need for region‐specific control strategies, including multipathogen vaccines and improved biosecurity.

## Graphical abstract

First comprehensive epidemiological and phylogenetic study of bovine respiratory disease complex (BRDC) in northern China (2022–2024). Dominant viral pathogens (BVDV, BPIV‐3) show winter seasonality and co‐infections with *Mycoplasma bovis*. Chinese isolates share evolutionary links with global strains, highlighting cross‐regional pathogen transmission for targeted control.

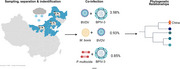

## 1 Introduction

One of the most serious diseases of the cattle industry worldwide is the bovine respiratory disease complex (BRDC), whose pathogenetic complexity and economic threat have been the focus of veterinary epidemiological research (Liu et al. 2022; Hao et al. 2024). Mixed infection of one or more pathogens between viruses and bacteria is characteristic of this disease (Lion et al. 2021). Its clinical manifestations, including fever, cough, pneumonia and death, burden the health of animals living in poor conditions and being transported frequently (Nissly et al. 2020; Zhou et al. 2023). More than half of dairy diseases worldwide have been believed to be related to BRDC, with high prevalence and low mortality (Ishida et al. 2020).

In North China, under the cold climate, high density of livestock and frequent transportation and trading, outbreaks of BRDC have been widespread. This study focused on six provinces in northern China: Inner Mongolia, Shanxi, Hebei, Liaoning, Heilongjiang and Shandong. These regions experience a temperate continental climate with pronounced seasonal variations. Winter spans from November to March, characterised by extreme cold average temperature: −20°C to −5°C and low humidity: 30%–50%. Summer, from June to August, is warm and humid, with temperatures ranging from 18°C to 25°C and relative humidity levels of 60%–80%. Such climatic extremes, combined with intensive livestock farming practices, may exacerbate respiratory pathogen transmission by prolonging viral stability in cold, dry air and increasing host susceptibility due to thermal stress. However, there are still gaps in the systematic epidemiological survey and phylogenetic research of pathogens in various regions (Liu et al. 2022).

This study aims to systematically investigate the prevalence, genetic diversity and co‐infection dynamics of BRDC pathogens in northern China through virus and bacterial isolation, molecular identification and phylogenetic analysis. Specifically, we focus on (1) characterising the dominant viral and bacterial pathogens in Holstein calves, (2) analysing their seasonal and regional distribution patterns and (3) reconstructing phylogenetic relationships between Chinese isolates and global strains. Phylogenetic analysis is critical to trace pathogen origins and transmission routes, identify emerging variants affecting vaccine efficacy and provide molecular evidence for transboundary disease monitoring—addressing gaps in regional BRDC control strategies. By addressing these gaps, this study provides region‐specific data to optimise vaccination strategies, biosecurity protocols and antimicrobial stewardship in northern China.

BRDC pathogens include two types of pathogens: viruses and bacteria (including *Mycoplasma*). Viruses are usually the primary source of infection, firstly attacking the respiratory mucosal barrier and promoting bacterial infection (Huang 2021; Bell et al. 2021). The principal viral pathogens are BHV‐1, BVDV, BRSV and BPIV‐3. The most prevalent bacterial pathogens (Bhattarai et al. 2022; Melchner et al. 2021) are *Mycoplasma bovis* (*M. bovis*), *Pasteurella multocida* (*P. multocida*) and *Mannheimia haemolytica* (*M. haemolytica*). In recent years, the dry infection rate of *M. bovis* has increased significantly, especially since the co‐infection with the virus has become particularly severe (Ozcan and Tutuncu, 2025). For instance, *M. bovis* co‐infection with BVDV can lead to toxicity and apoptosis of bovine macrophages (Bomac cells) (Bürgi et al. 2018).

Synergy mechanisms of pathogens are a priority research area of BRDC (El‐Mayet et al. 2022). For instance, BRSV infection allows the expression of adhesion molecules on the surface of the respiratory cell, which allows *P. multocida* to colonise quickly (Sudaryatma et al. 2018). On the other hand, the latent infectious character of BHV‐1 enables viral reactivation during stress, which facilitates the secondary bacterial infections in cattle (El‐Mayet and Jones, 2024). In addition, double bacterial infections (e.g., *Pasteurella* vs. *Mycoplasma*) may amplify lung damage through interactions of their virulence factors (Gaudino et al. 2022). These results imply that prevention and control of BRDC can shift from addressing a single pathogen to treating the issue of multi‐pathogen interactions.

Currently, vaccination against BRSV in northern China is not widely implemented in most small‐scale farms due to economic constraints and fragmented veterinary services. However, some large‐scale dairy operations have adopted routine BRDC vaccination programs for calves, primarily using inactivated or subunit vaccines. Despite these efforts, the coverage remains inconsistent and vaccine efficacy may be compromised by field strain heterogeneity.

This research is the first large‐scale isolation and identification of BRDC pathogens in northern China. Combining pathogen isolation, identification, sequencing and phylogenetic analysis, the results of this study will provide data to support the development of regionalised BRDC prevention and control programmes and give a theoretical basis for understanding the molecular mechanisms of multi‐pathogen co‐infection.

## 2 Materials and Methods

### 2.1 Sample Collection and Processing

All experimental protocols were approved by the Key Laboratory for Clinical Diagnosis and Treatment of Animal Diseases at the Hohhot Inner Mongolia Agricultural University (Approval number NND2021108, 20 December 2021). All methods described in this article were performed in accordance with the relevant ethical guidelines and regulations. From January 2022 to December 2024, we analysed 5052 samples on Holstein calves (5–10 months old) with BRDC symptoms in northern China. The 5052 samples were aseptically collected, each calf contributed a single sample type, including nasal swabs (*n =* 3347), serum (*n =* 1423) and post‐mortem lung/tracheal tissues (*n =* 282). Nasal swabs were immersed in transport mediums (DMEM). Samples were transported to the laboratory on dry ice within 2 h of collection and stored at −80°C for long‐term storage. Lung/tracheal tissues were cryosectioned into 1 cm^3^ cubes, homogenised via cryogenic milling (Retsch MM400) and stored in liquid nitrogen. Serum was filtered (0.22 µm PVDF membrane), aliquoted and flash‐frozen at −80°C.

### 2.2 Isolation and Culture of Bacteria and Viruses

After the pretreatment described in Section 2.1, a sterile inoculation ring was used to inoculate freshly prepared whole blood agar media with the tissue and nasal swab samples. One sample with solid medium was placed in an anaerobic tank at 37°C and the other in a constant temperature incubator at 37°C. After 24 h of culture, the colony characteristics were observed and use a sterile inoculation needle to pick up half of a single colony for Gram staining, observe the morphology of bacteria under a microscope, then pick up the other half of the same colony and inoculate it into the nutrient broth medium and culture at 37°C for subsequent experiments.

In the meantime, the pre‐treated bovine nasal swab and tissue samples from Section 2.1 were spread on PPLO solid medium (BD Biosciences, New Jersey, USA) containing 5% yeast extract (Thermo Fisher Scientific, Altrincham, UK), 20% horse serum (HyClone, Logan, USA), 200 IU/mL penicillin, 0.125 mg/mL sodium pyruvate (Sangon Biotech, Shanghai, China) and 2% agar (Hopbiol, Qingdao, China) and cultured at 37°C under 5% CO_2_ for 5–7 days. The colonies were observed using a light microscope. A single colony was picked with an inoculum needle, placed in 3 mL PPLO liquid medium and cultured at 37°C under 5% CO_2_ for 5–7 days for follow‐up experiments.

The collected bovine nasal swabs, tissue and serum samples were inoculated into three bottles of monolayer Madin‐Darby bovine kidney (MDBK) cells (China Academy of Sciences Cell Bank, Shanghai, China) and 1% double antibody (BD Biosciences, New Jersey, USA) was added into culture‐medium, then incubated at 37°C for 2 h. The basic DMEM culture medium was added (3 mL) and the cytopathic effect (CPE) was observed every 24 h for 5 days, the culture was collected when 80% of cells occur the CPE. The MDBK cell cultures infected with BHV‐1, BVDV, BPIV3 and BRSV were collected, frozen and thawed at −80°C and room temperature 23–25°C three times and centrifuged at 1000 rpm for 10 min to remove cell fragments and collect the supernatant for PCR analysis.

### 2.3 Molecular Identification

Nucleic acids were extracted from serum, bacterial cultures, *M. bovis* media and viral lysates, using the DNA/RNA Extraction Kit (TIANGEN, China) following the manufacturer's protocol. Briefly, samples were lysed with Buffer RL and centrifuged at 12000 × g for 1 min. DNA/RNA was eluted in 50 µL of Elution Buffer and stored at –80°C until further analysis. For RNA viruses (BVDV, BRSV, BPIVn3), reverse transcription was performed using the TransScript One‐Step RT‐PCR SuperMix, according to the manufacturer's instructions. RNA (8 µL) was mixed with 2 µL of 5 × TranScript II All‐in‐One SuperMix, incubated at 50°C for 15 min, followed by inactivation at 85°C for 5 s. cDNA was stored at –20°C prior to RT‐PCR amplification. PCR amplification (25 µL system) targeted: (1) BHV‐1 gD gene (MG407792.1), (2) bacterial 16S rRNA V3–V4 regions, (3) *M. bovis* PpSM gene, (4) *P. multocida* KMT1 gene and (5) *M. haemolytica* gcp gene. RT‐PCR (TaKaRa one‐step system) detected BVDV (5'‐UTR; XM_040428314.1), BRSV (N gene; NC_038272.1) and BPIV3 (M gene; NC_006430.2). All primers (maintained in this laboratory) are shown in Table 1. Cycling conditions: 95°C (5 min), 35 cycles of 95°C/30 s, target‐specific annealing/45 s, 72°C/1 min/kb and final extension (72°C/10 min). Amplicons were electrophoresed (1.5% agarose, 100 V/30 min) and target DNA bands were excised and purified using the DNA Gel Extraction Kit (Axygen, USA) according to the manufacturer's protocol. The purified DNA was sequenced bidirectionally via sanger sequencing and phylogenetically analysed (MEGA‐X, neighbour‐joining with 1000 bootstrap replicates).

**TABLE 1:** The primer‐probe sets used for the detection system.

Target pathogen	Primer Sequence (5′‐3′)	Target gene	Product size (bp)	TM (°C)
BVDV	F: ATGCCCTTAGTAGGACTAG R: TCAACTCCATGTGCCATGT	5'‐UTR	287	53
BHV‐1	F: GCTCGCCAACTTCTTTCAGGG R: GCGTCAAACTCCTCCTCTTCCTC	gB	306	60
BPIV3	F: AGTGATCTAGATGATGATCCA R: GTTATTGATCCAATTGCTGT	M	329	49
BRSV	F: ATGGCTCTTAGCAAGGTC R: AGAGTCATGTCTGTATTC	N	459	50
*P. multocida*	F: ATCCGCTATTTACCCAGTGG R: GCTGTAAACGAACTCGCCAC	KMT1 (Townsend et al., 1998)	460	50
*M. haemolytica*	F: TGGGCAATACGAACTACTCGGG R: CTTTAATCGTATTCGCAG	gcp (Shanthalingam et al., 2014)	227	55
*M. bovis*	F: CCAGCTCACCCTTATACATGAGCGC R: TGACTCACCAATTAGACCGACTATTTCAC	PpSM	442	60

### 2.4 Pathogen Characterisation

Bacterial biochemical reactions were determined using commercial biochemical test tubes containing glucose, fructose, lactose, sucrose and mannitol (Guangzhou Huankai Microbial Technology Co., Ltd., China). The tubes were incubated at 37°C for 48 h. The reactions were interpreted according to the manufacturer's instructions (colour change for acid production and bubbles for gas production). *P. multocida* and *M. haemolytica* were biochemically profiled. We examined virus cultures showing CPE and PCR positivity using transmission electron microscopy (TEM): lysates centrifuged (12,000 × *g*/10 s), inactivated (56°C/30 min) and negatively stained with 2% phosphotungstic acid (pH 6.8) on Formvar‐coated grids and imaged the virion structure (JEM‐1400 TEM, 80 kV).

### 2.5 Data Analysis

Statistical analyses were performed using IBM SPSS Statistics for Windows, Version 28.0 (IBM Corp., Armonk, NY, USA; released 2021). Surveillance data spanning 2022 to 2024 were aggregated quarterly and pathogen‐specific positivity rates were calculated as the proportion of pathogen‐positive samples relative to the total samples tested. Temporal trends were assessed through descriptive statistics, with categorical variables (e.g., seasonal variations in pathogen detection rates) analysed using the Chi‐square test. Continuous variables (e.g., annual prevalence trends) were compared via one‐way analysis of variance (ANOVA).

## 3 Results and Analysis

### 3.1 Bacterial Isolation and Phenotypic Characterisation

**FIGURE 1:**
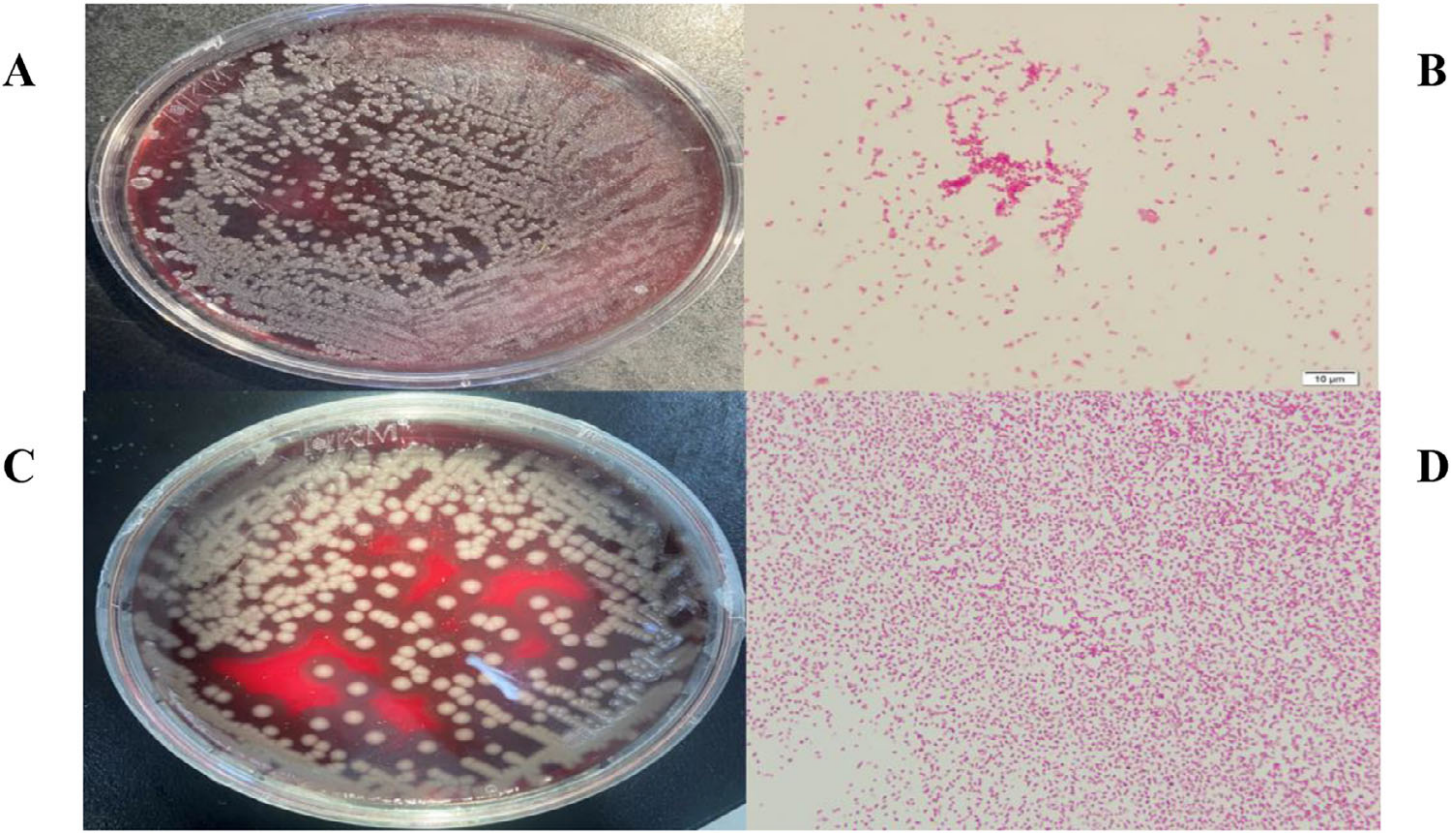
Colonies and Gram stain images of *M. haemolytica* and *P. multocida*. (A) The colony morphology of *M. haemolytica* on the plate, (B) the morphology of the colony under a microscope after Gram staining (scale: 10 µm), (C) the colony morphology of *P. multocida* on the plate, and (D) the morphology of the colony under a microscope after Gram staining (scale: 10 µm).

**FIGURE 2:**
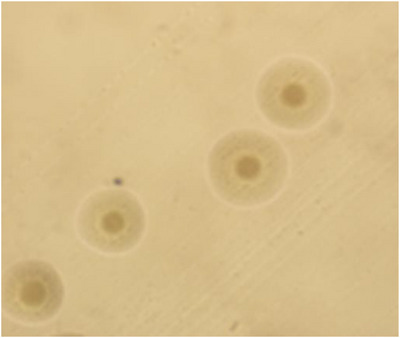
Colonies with typical *M. bovis* morphology (scale: 100 µm).

*M. haemolytica* and *P. multocida* were identified as primary bacterial pathogens following the standard bacteriological protocols (Section 2.2). *M. haemolytica* colonies exhibited smooth morphology on blood agar with weak β‐haemolysis (Figure 1A), while *P. multocida* formed convex colonies with bipolar staining characteristics (Figure 1C). Gram staining confirmed both species as Gram‐negative bacilli (Figure 1B,D). *M. bovis* displayed the distinctive “fried egg” morphology on PPLO agar (Figure 2), consistent with its fastidious growth requirements.

### 3.2 Viral Isolation and Cytopathic Effects

**FIGURE 3:**
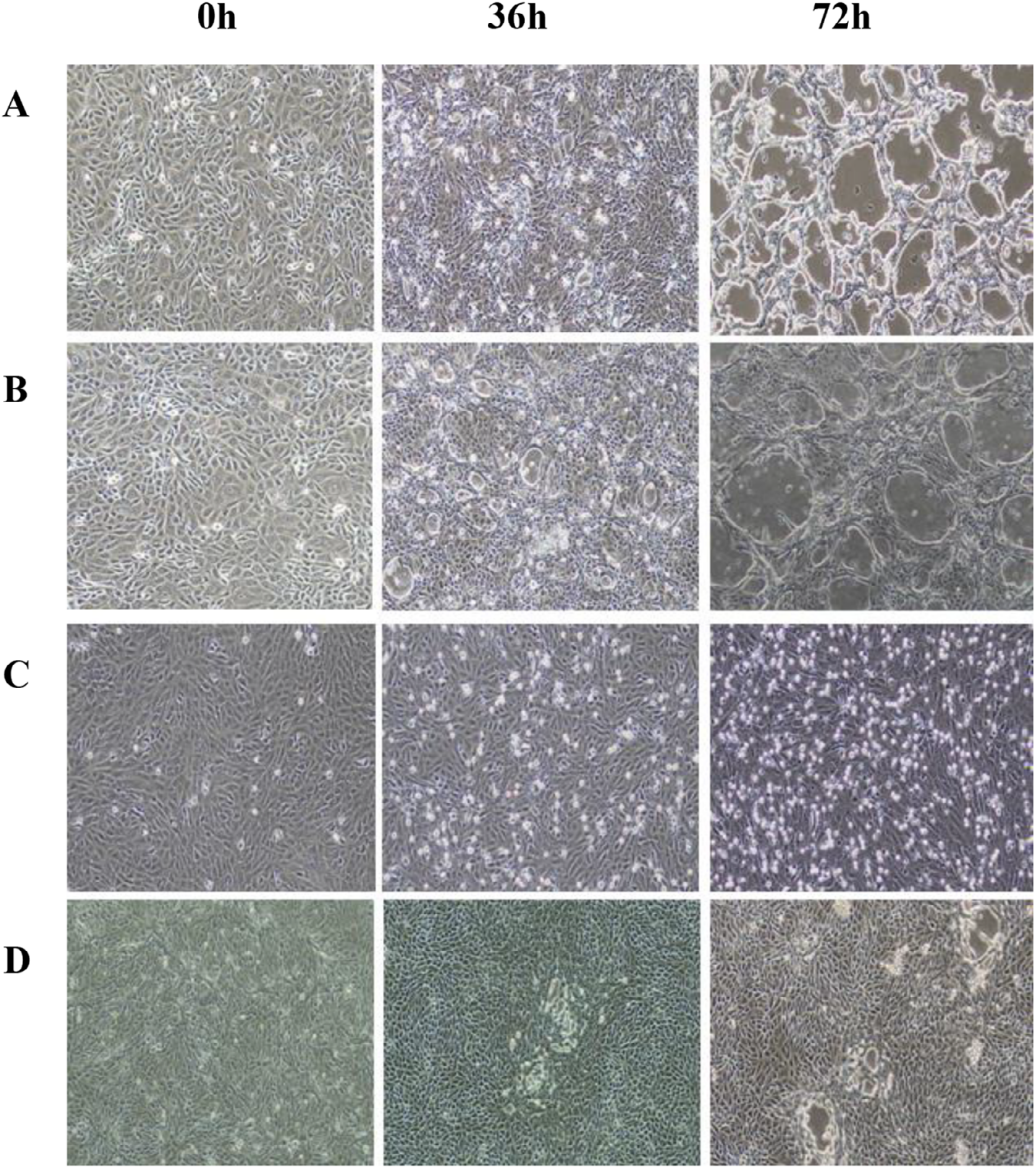
CPE of MDBK cells infected with different viruses. (A) The cytopathic changes in BVDV‐infected cells at 0, 36, and 72 h under an optical microscope, (B) the cytopathic changes in BHV‐1‐infected cells at 0, 36, and 72 h under an optical microscope, (C) the cytopathic changes in BPIV‐3‐infected cells at 0, 36, and 72 h under an optical microscope, (D) the cytopathic changes in BRSV‐infected cells at 0, 36, and 72 h under an optical microscope.

Viral propagation in MDBK cells (Section 2.2) revealed distinct cytopathic patterns: BVDV, non‐cytopathic biotypes predominated (72% of isolates), inducing vacuolation and cell detachment by 72 h post‐inoculation (Figure 3A); BHV‐1, rapid cell fusion and vacuolation were observed within 36 h (Figure 3B); BPIV‐3, syncytia formation became evident by 72 h (Figure 3C); BRSV, cell rounding and fusion occurred as early as 36 h (Figure 3D).

### 3.3 Biochemical and Molecular Identification

**FIGURE 4:**
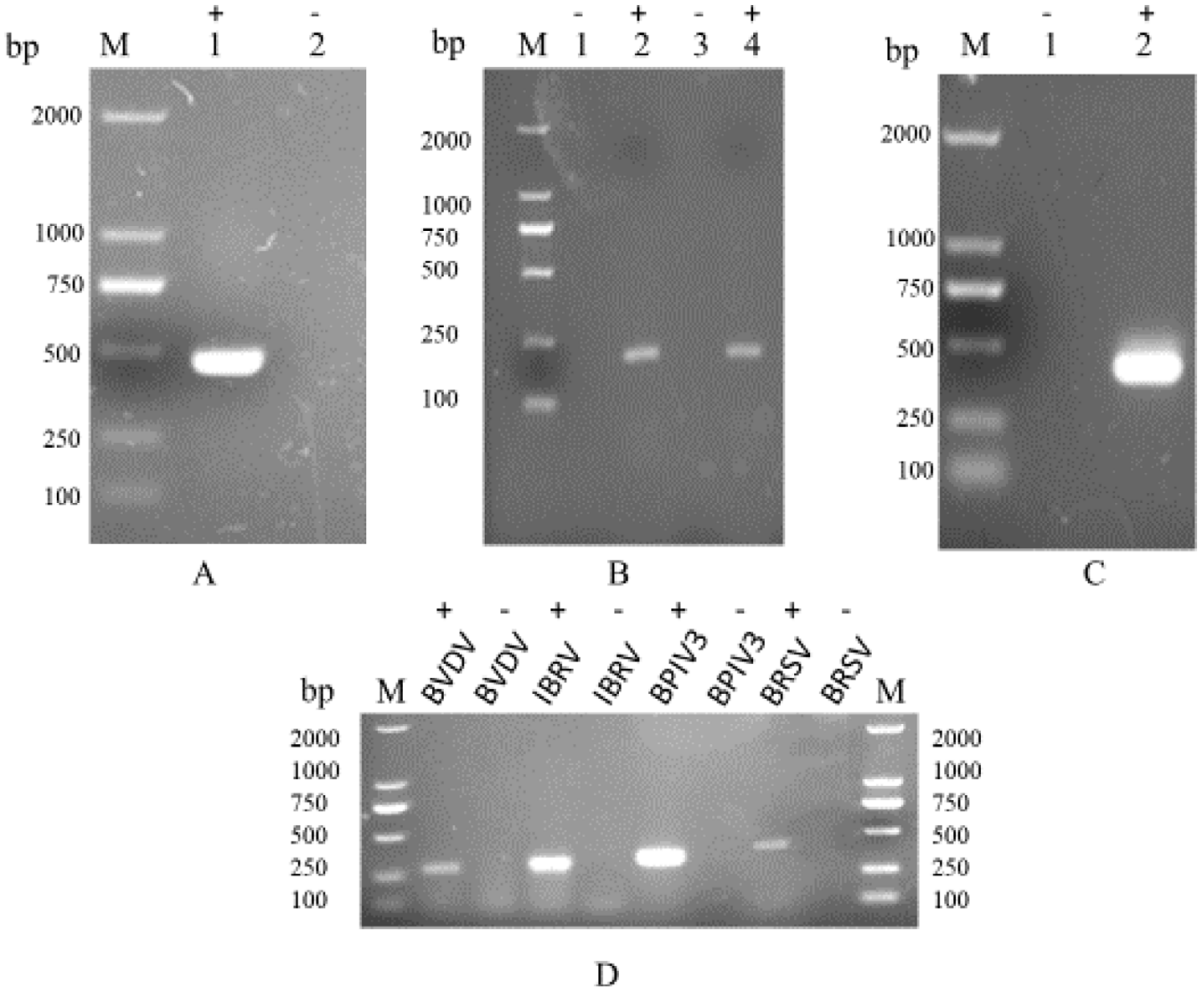
The results of PCR identification. “+” means positive; “−” means negative.(A) The gel electrophoresis band of *P. multocida*, (B) the gel electrophoresis band of *M. haemolytica*, (C) the gel electrophoresis band of *M. bovis*, and (D) the electrophoresis bands of BVDV, BHV‐1, BPIV‐3, and BRSV from left to right.

Biochemical profiling (Section 2.4) differentiated *P. multocida* (glucose‐positive, sucrose‐positive, indole‐positive) from *M. haemolytica* (lactose‐positive, mannitol‐negative) (Table 2). PCR amplification (Section 2.3) yielded pathogen‐specific bands: Bacterial targets: *P. multocida* (approximately 460 bp), *M. haemolytica* (approximately 227 bp) and *M. bovis* (approximately 442 bp) (Figures 4A–C); viral targets: BVDV (approximately 287 bp), BHV‐1 (approximately 306 bp), BPIV‐3 (approximately 328 bp) and BRSV (approximately 459 bp) (Figure 4D). Sequencing validation confirmed amplified products exhibited high concordance with conserved regions of target pathogens.

**TABLE 2:** Biochemical test results of *P. multocida* and *M. haemolytica*.

Items	*P. multocida*	*M. haemolytica*
Glucose	+	+
Maltose	−	+
Lactose	+	+
Sucrose	−	−
Mannitol	+	−
Indole test	+	−

*Note*: “+” means positive; “−” means negative.

### 3.4 Phylogenetic Relationships

**FIGURE 5:**
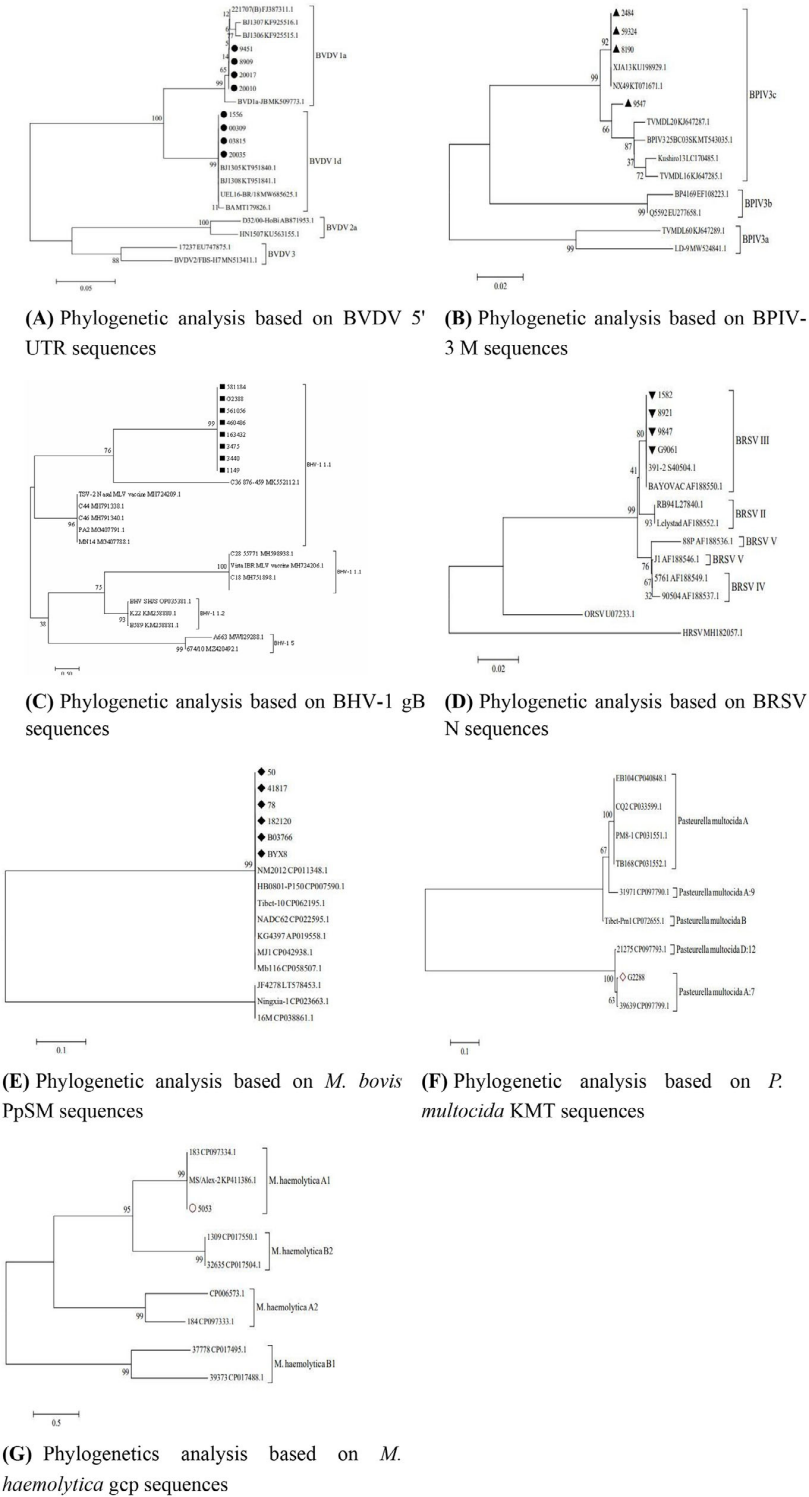
Phylogenetic analysis of sequenced isolated strains. (A) Phylogenetic analysis based on BVDV 5**′** UTR sequences (samples of BVDV are marked with solid black circles); (B) phylogenetic analysis based on BPIV‐3 M sequences (samples of BPIV‐3 are marked with solid black triangle); (C) phylogenetic analysis based on BHV‐1 gB sequences (samples of BHV‐1 are marked with solid black squares); (D) phylogenetic analysis based on BRSV N sequences (samples of BRSV are marked with solid black inverted triangle); (E) phylogenetic analysis based on *M. bovis* PpSM sequences (samples of *M. bovis* are marked with solid black rhombus); (F) phylogenetic analysis based on *P. multocida* KMT sequences (samples of *P. multocida* are marked with red hollow rhombus); (G) phylogenetic analysis based on *M. haemolytica* gcp sequences (samples of *M. haemolytica* are marked with red hollow circle).

Phylogenetic reconstruction (Section 2.3) showed the following: BVDV subgenotypes 1a (*n =* 4, strains: 20010, 9451, 8909, 20017) and 1d (*n =* 4, strains: 2484, 59324, 8190, 9547) were closely related to North American/European stains (Figure 5A); BPIV‐3 (*n =* 4, strains: 2484, 59324, 8190, 9547) formed a monophyletic group with Turkish BPIV‐3c strains (Figure 5B);  BHV‐1 (*n =* 8, strains: 581184, G2388, 56456, 460486, 163432, 3475, 3440, 1149) were clustered within the same branch as the reference strain C36876‐459 and were classified into subtype BHV‐1.1 (Figure 5C); BRSV (*n =* 4, strains:1582, 8921, 9847, G9061) were classified into genotype III (Figure 5D); *M. bovis* (*n =* 6, strains: 50, 41817, 78, 182120, B03766, BYX8) exhibited > 98.7% homology with global reference strains (Figure 5E). *P. multocida*: a representative isolate (strains: G2288) based on KMT1 gene sequencing was clustered with the reference strain 39639 and was classified as type A7 (Figure 5F). *M. haemolytica*: one strain (strain: 5053) analysed via gcp gene alignment showed 99.2% homology with reference strains 183 (GenBank: KY242518.1) and MS/Alex‐2 (GenBank: CP047557.1), and was classified as serotype A1 (Figure 5G).

### 3.5 Viral Ultrastructural Features

**FIGURE 6:**
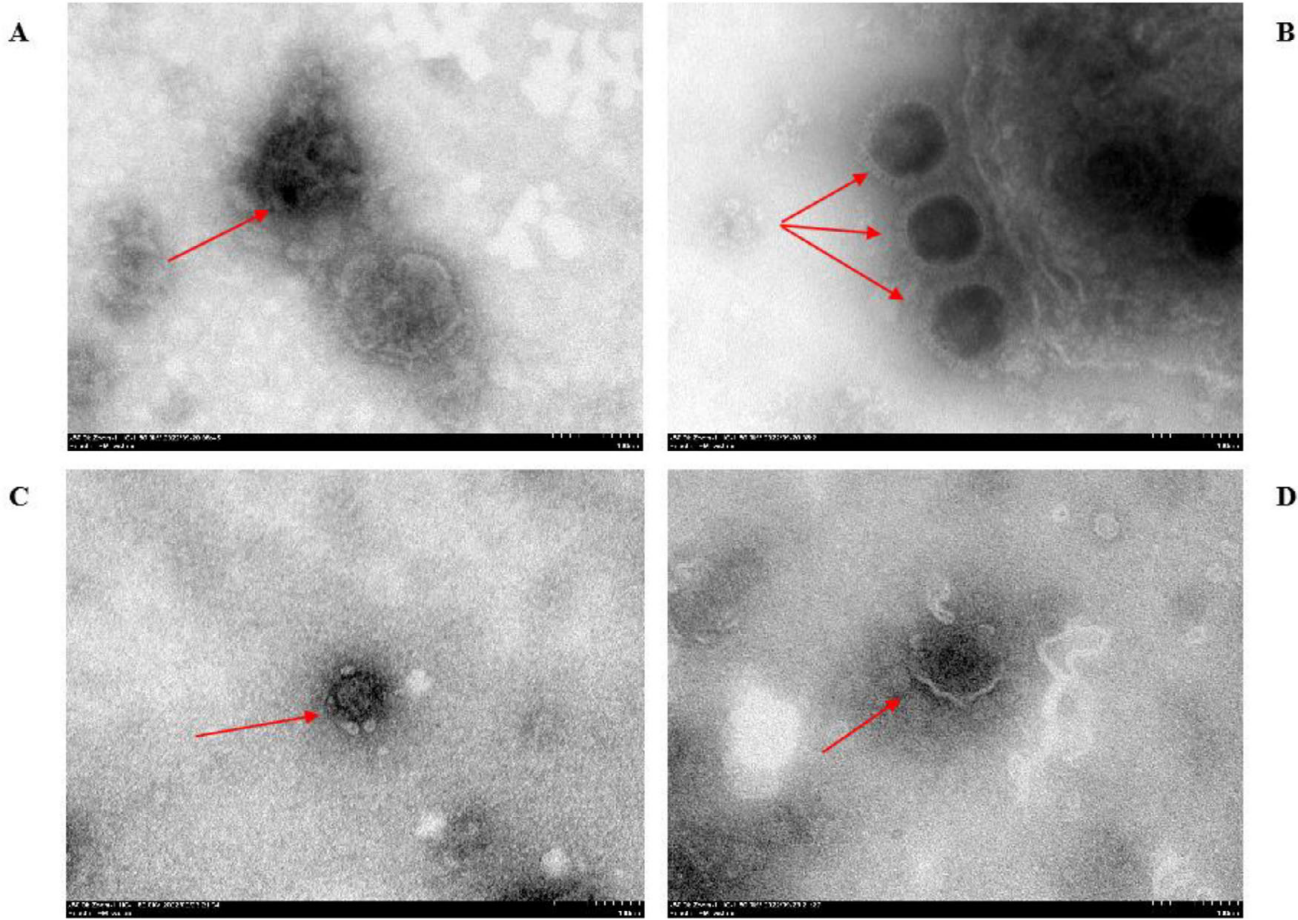
Transmission electron microscopy images of four virus. (A) Morphology of BPIV‐3 virus particles under transmission electron microscopy; (B) morphology of BHV‐1 virus particles under transmission electron microscopy; (C) morphology of BVDV virus particles under transmission electron microscopy; (D) morphology of BRSV virus particles under transmission electron microscopy (JEM‐1400 TEM, all scales are 100 nm).

Transmission electron microscopy (Section 2.4) resolved virion morphologies—BVDV: spherical particles measuring 60 ± 5 nm in diameter (Figure 6C); BHV‐1: enveloped virions with diameters of 120 ± 10 nm (Figure 6B); BPIV‐3: enveloped particles bearing surface projections (180 ± 15 nm) (Figure 6A); BRSV: spherical structures (80 ± 5 nm) (Figure 6D).

### 3.6 Epidemiological Trends

**FIGURE 7:**
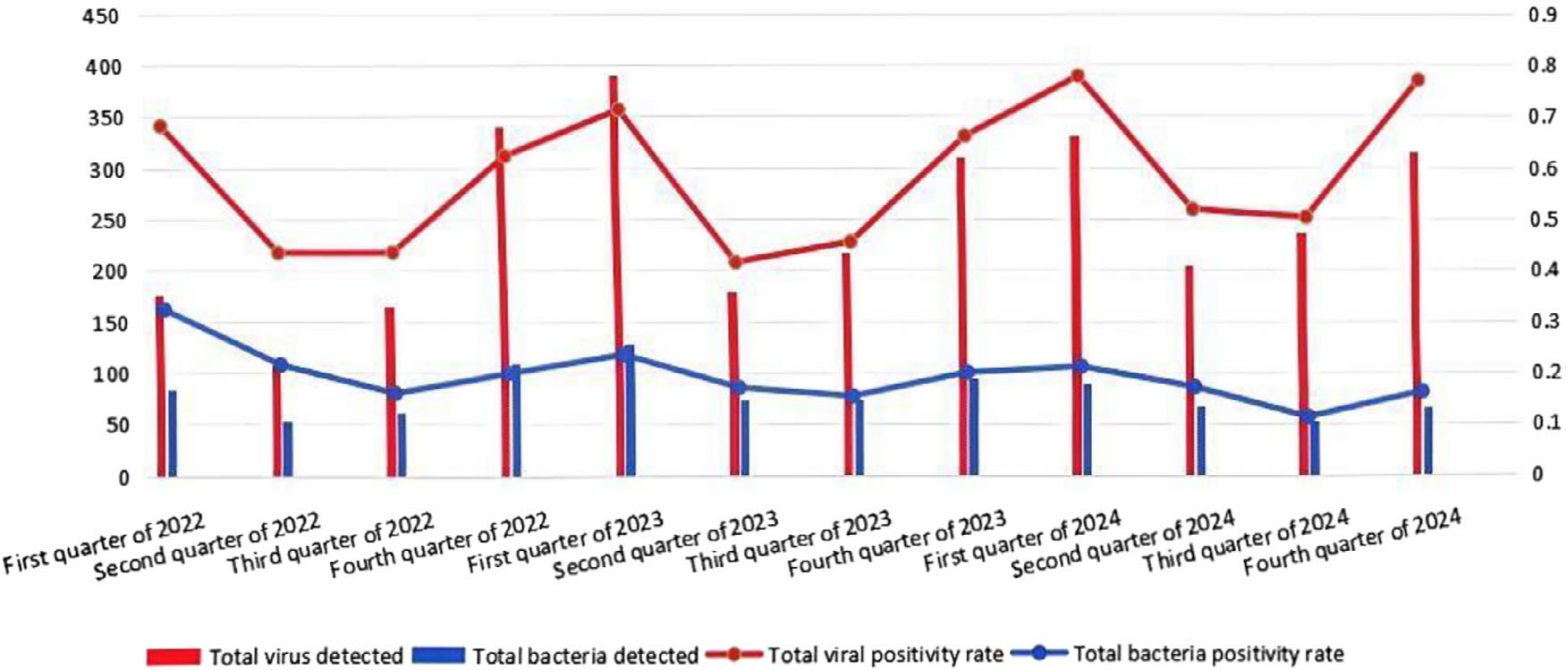
Comparing viral/bacterial positives and positive rates across quarters.

**FIGURE 8:**
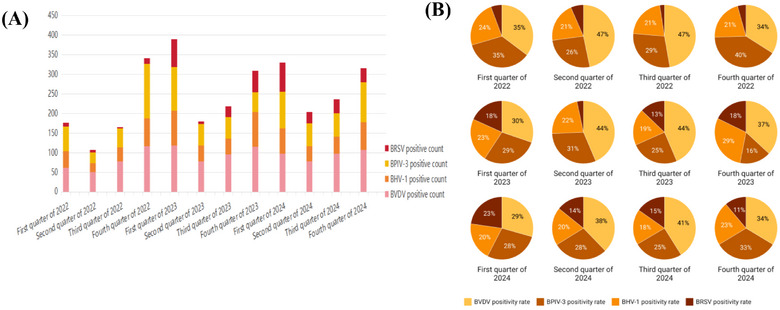
Quarterly distribution of viral detection. (A) Number of positive samples per pathogen. (B) Positivity rates (%) for each viral pathogen across quarters.

**FIGURE 9:**
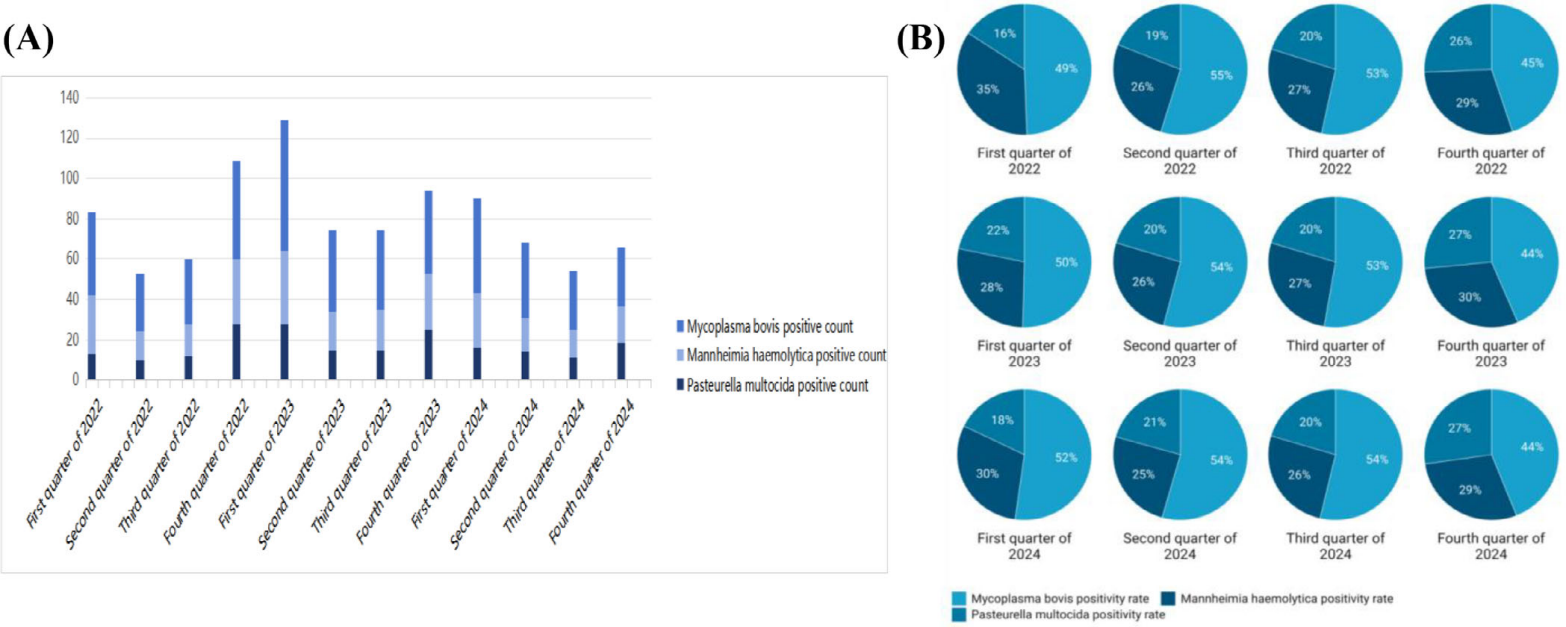
Quarterly distribution of viral detection. (A) Number of positive samples per pathogen. (B) Positivity rates (%) for each bacterial pathogen across quarters.

In this epidemiologic survey, the total number of viral positives was 2974 and the total number of bacterial positives was 953. The quarterly average prevalence rates were BVDV (21.58%), BHV‐1 (12.73%), BPIV‐3 (16.94%), BRSV (6.98%), *P. multocida* (4.04%), *M. haemolytica* (5.54%) and *M. bovis* (9.74%). Statistical analyses (Section 2.5) revealed—Viral seasonality: significantly elevated detection rates in Q1 (72.85%, *n =* 965/1425) and Q4 (67.72%, *n =* 896/1230) (*χ* ^2^ = 54.18, *p <* 0.001; Figure 7), the *p*‐value analysis based on the chi‐square test yielded highly significant differences in infection rates of individual viruses between quarters; Annual dynamics: BVDV prevalence declined from 21.55% (2022) to 20.48% (2024) (ANOVA: *F* = 2.883, *p =* 0.1027), whereas BRSV increased from 2.63% to 10.3% (*F* = 1.539, *p =* 0.2778) (Figure 8B), according to a one‐way ANOVA, there was no difference in the prevalence of infection for each virus between years; Bacterial seasonality: seasonal variation in the prevalence of three bacterial infections was not significant (*χ* ^2^ = 39.19, *p =* 0.21; Figure 9B); Annual dynamics: *M. bovis* maintained dominance (mean prevalence: 9.74%, *F* = 6.098, *p =* 0.0183), prevalence of *P. multocida* (F = 9.236, *p =* 0.0056) ranged from 2.35% to 5.34% and *M. haemolytica* (*F* = 4.291, *p =* 0.0442) ranged from 2.99% to 11.24%. There was no difference in the prevalence of infection for each bacterium between years.

### 3.7 Co‐Infection Dynamics

Analysis of mixed infections (Table 3) demonstrated: viral–viral co‐infections predominated, notably BVDV/BPIV‐3 (3.98%, *n =* 201/5052) and BVDV/BHV‐1 (2.63%, *n =* 133/5052); viral–bacterial interactions were less frequent, exemplified by BHV‐1/*M. bovis* co‐detection (1.58%; *χ* ^2^ = 67.2, *p <* 0.001).

**TABLE 3:** The co‐infection prevalence of different pathogens.

Co‐infection with different pathogens	Positive(n)/total case	Prevalence (%)
BVDV/BRSV	79/5052	1.56
BVDV/BHV‐1	133/5052	2.63
BVDV/BPIV‐3	201/5052	3.98
BRSV/BHV‐1	132/5052	2.61
BRSV/BPIV‐3	151/5052	2.99
BPIV‐3/ *P. multocida*	43/5052	0.85
BPIV‐3/ *M. bovis*	45/5052	0.89
BVDV/ *M. bovis*	47/5052	0.93
BHV‐1/ *M. bovis*	80/5052	1.58
BRSV/ *M. bovis*	43/5052	0.85
BPIV‐3/BHV‐1/BVDV	43/5052	0.85

### 3.8 Prioritisation of Vaccine Targets Based on Prevalence, Co‐Infection and Subtype Assessment

To translate epidemiological findings into actionable interventions, we evaluated vaccination priorities based on pathogen prevalence, co‐infection burden and subtype distribution (Table 4). BVDV and BPIV‐3 emerged as highest‐priority targets due to their high co‐occurrence, dominance in multi‐pathogen infections and available vaccines matching regional subtypes (1a/1d and 3c).

**TABLE 4:** Evidence‐based vaccination priorities for BRDC in northern China.

Pathogen	Prevalence	Co‐infection role	Regional subtype	Vaccine priority	Rationale
BVDV	21.58%	Dominant in 63% of co‐infections (Table 3)	1a/1d (Figure 5A)	Highest	High prevalence + predominantly non‐cytopathic (72%) → difficult to diagnose
BPIV‐3	16.94%	Key partner in BVDV/BPIV‐3 (3.98%, Table 3)	Genotype 3c (Figure 5B)	High	Strong association with BVDV
*M. bovis*	9.74%	Primary bacterial co‐infectant (58.3% virus‐bacteria co‐infections, New Table 4)	Global strains (Figure 5E)	High	Highest prevalence among bacteria + Lack of effective vaccines
BHV‐1	12.73%	Latency‐triggered outbreaks (Line 100–102)	Subtype 1.1 (Figure 5C)	Moderate	Stress‐induced reactivation; commercial vaccines available
BRSV	6.98%	Increasing trend (2.63%→10.3%, Figure 8B)	Genotype III (Figure 5D)	Contextual	Prioritise in large‐scale farms

## 4 Discussion

BRDC remains a vital global cattle industry issue, with its multifactorial aetiology and economic repercussions demanding region‐specific epidemiological insights (Zhou et al. 2023; Ferraro et al. 2021). This study provides the first comprehensive investigation of BRDC pathogen diversity, transmission dynamics and phylogenetic relationships in northern China, a region characterised by high livestock density, climatic extremes and frequent interprovincial cattle movements. The study's results deeply reveal the pathogens’ individual and collective infection status and emphasise the evolutionary relationship between heterogeneous strains, theoretically maximising the local control measures.

The BRDC epidemiology in North China has precise regional and seasonal distribution. The antibody‐positive rate of BRSV in large‐scale dairy farms of four provinces in North China was 67.4%, the BHV‐1 infection rate exceeded 50% and the rate of mixed infection was higher with the increasing animal trading intensity and breeding density (Xu et al. 2024; Bi et al. 2020). In Inner Mongolia's low temperature, especially the closed breeding room in winter, the incidence of BRDC was 2.3 times higher in winter than in summer. The mixed infection of pathogens is showing an upward trend: virus mixed infection (such as BHV‐1 + BRSV) accounts for 42% and virus bacteria mixed infection (such as BRSV+*M. haemolytica*) accounts for 38% (Bi et al. 2020).

Separating and identifying viral and bacterial pathogens exhibited a complex primary and second agent interaction. Consistent with global trends, BVDV, BRSV, BPIV‐3 and BHV‐1‐associated‐viral pathogens dominated the BRDC cases, typically compromising breathing defences (Fulton 2020; Kamel et al. 2024). The viral coinfection phenomenon exhibits a high detection rate among multi‐pathogen infection cases. Pathogenesis of BRDC has been proven by the synergistic form of *M. bovis* infection with other pathogens (Lion et al. 2021).According to studies, the prevalence of this pathogen in mixed infections has come close to that of the prevailing respiratory infections and herd surveillance studies in geographical locations have supported this epidemiological observation (Buczinski et al. 2024). Synergistic pathogenic mechanisms (e.g., enhanced adherence of *M. haemolytica* to lower respiratory epithelium) have been demonstrated by BHV‐1‐mediated upregulation of host cell adhesion molecule expression (Cowick et al. 2022). Our findings emphasise that pathogen ecology‐based comprehensive strategies that are more efficient than single‐pathogen targeting regimens are required to address present respiratory disease problems in the livestock sector.

The polymicrobial nature of BRDC observed in northern China aligns with global patterns. A recent UK study (Denholm et al. 2024) similarly reported 77.6% of clinical samples harboured multiple bacterial species and 17.7% contained multiple viruses, reinforcing that co‐infections dominate BRDC presentations worldwide. Notably, both studies identified* M. bovis* as a central player in mixed infections—significantly associated with *P. multocida* and *M. haemolytica* in our cohort (Table 3) and with *P. multocida/Histophilus somni* in the UK cohort. This conserved pattern across geographically distinct cattle populations underscores the need for control strategies targeting pathogen synergies rather than individual agents.

Its high seasonality in peaks in Q1 and Q4 (Figure 7), combined with cold stress and compromised ventilation in north Chinese winter, is favourable to the transmissions of respiratory pathogens. This has been supported by evidence attributing low temperatures to viral‐enhanced stability and host immunosuppression (Aboubakri et al. 2022). Bacterial positivity rates remained stable year‐round, suggesting environmental persistence and management‐related factors (e.g., transport stress) as key contributors. The 2.3‐fold higher BRDC incidence in winter versus summer (Inner Mongolia cohort) mirrors findings from temperate regions (Buczinski et al. 2021), reinforcing the need for seasonal vaccination protocols and improved husbandry practices.

Phylogenetic analysis revealed significant evolutionary relationships between Chinese isolates and international strains. For instance, BVDV subgenotypes 1a and 1d identified in this study (Figure 5A) correspond to lineages prevalent in North America and Europe (Yeşilbağ et al. 2017), highlighting the global circulation of BRDC pathogens. The BPIV‐3c subtype dominance (Figure 5B) aligns with recent reports from Turkish (Muftuoglu et al. 2021), indicating convergent evolutionary pressures. Regarding BRSV, phylogenetic classification into genotype III aligns with previous reports from Chinese isolates (Xu et al. 2024; Zhou et al. 2023), which predominantly clustered within this lineage. However, compared to historical strains from northern China (Bi et al. 2020), the current BRSV isolates exhibited a 2.1% divergence in the N gene sequence, potentially indicating localised evolutionary adaptation. For BHV‐1, the gB gene sequences showed > 99% homology with domestic strains reported by Liu et al. (2022), suggesting genetic stability of BHV‐1 in this region. Notably, no novel mutations associated with immune evasion were observed, contrasting with emerging BHV‐1 variants in Europe (Bell et al. 2021). These findings underscore the importance of international collaboration in pathogen surveillance and vaccine development. The high detection rates of BVDV and BPIV‐3 via RT‐qPCR validate the utility of molecular diagnostics in BRDC management. However, non‐cytopathic BVDV strains dominated, complicating timely diagnosis, necessitating serological screening to identify persistently infected carriers (Ma et al. 2022). The decline in *M. bovis* detection from 2022 to 2024 (Figure 9B) may reflect improved farm biosecurity, yet its persistent prevalence warrants targeted antimicrobial stewardship to mitigate resistance. Furthermore, the identification of *P. multocida* type A7, a serotype associated with severe pneumonic lesions (Getnet et al. 2022), calls for region‐specific bacterin formulations. While this study focused on classical BRDC pathogens, recent evidence suggests emerging viruses like influenza D virus (IDV) may contribute to respiratory complexes in northern China. Studies in North American cattle (Saegerman et al. 2022) reported IDV co‐detection with BRSV and *M. bovis* in 5.3% of BRDC cases, with phylogenetic analyses indicating reassortment between distinct clades. Although not investigated here, future surveillance should include IDV given its confirmed role in bovine respiratory disease and potential interactions with dominant pathogens identified in this study.

Moreover, our findings on the elevated prevalence of antimicrobial resistance (AMR) in bacterial pathogens such as M. bovis align with recent North American surveillance data. Andrés‐Lasheras et al. (2021) reported that beef cattle sourced from backgrounding operations exhibited significantly higher odds of harbouring AMR bacteria (e.g., multidrug‐resistant Pasteurellaceae) compared to auction‐derived calves, likely due to prolonged antimicrobial exposure in confined pre‐feedlot environments. This underscores the need to integrate management practices (e.g., minimising commingling, optimising antimicrobial stewardship in backgrounding systems) alongside vaccine development in northern China's BRDC control strategies. Such measures may mitigate the emergence of AMR strains complicating BRD therapy.

While this study provides critical baseline data, several limitations merit consideration. First, longitudinal studies tracking individual animals from birth to slaughter could elucidate temporal infection dynamics. Second, metagenomic sequencing was not employed to detect unculturable or novel pathogens, potentially underestimating BRDC complexity. Future work should integrate next‐generation sequencing to uncover underrepresented agents. Third, the focus on Holstein calves may limit generalisability to other breeds or age groups. Expanding surveillance to include beef cattle and adult dairy cows would enhance epidemiological relevance.

Therefore, in our future research planning, we will focus on the mechanism of synergistic infection among pathogens of BRDC and the development of novel and efficient vaccines.

## 5 Conclusion

This study delineates the etiological panorama of BRDC in northern China, emphasising the relationship between viral and bacterial pathogens, climatic and management‐associated stressors and global pathogen mobility. The phylogenetic relationships to international strains underscore the necessity for transboundary disease monitoring, while the seasonal infection patterns encourage responsive management strategies. By integrating these findings into regional vaccination programs, biosecurity protocols and antimicrobial guidelines, stakeholders can reduce BRDC's economic and welfare impacts. Future research should focus on multivalent vaccines for dominant subgenotypes and investigate host genetic factors influencing BRDC susceptibility.

## Author Contributions

**Yaping Zhou**: conceptualisation; methodology, formal analysis, investigation, writing – original draft, writing – review and editing. **Ting Guo**: methodology, investigation. **Hongmei Zhao**: methodology, investigation. **Yongqing Hao**: conceptualisation, fund acquisition. All the authors have read and agreed to the published version of the manuscript.

## Ethics Statement

Before using the animals to collect all samples, we contacted the owners of each cattle farm and obtained their permission. The owners also agreed to the use and disclosure of the data generated by this study. All experimental protocols were approved by the Key Laboratory for Clinical Diagnosis and Treatment of Animal Diseases at the Hohhot Inner Mongolia Agricultural University (Approval number NND2022108, accessed on 20 December 2022). All the methods described in this article were performed in accordance with the relevant ethical guidelines and regulations.

## Conflicts of Interest

The authors declare no conflicts of interest.

## Peer Review

The peer review history for this article is available at https://www.webofscience.com/api/gateway/wos/peer‐review/10.1002/vms3.70608.

## Data Availability

All the data analysed during this study are included in this article. Some of the nucleotide sequenced data have been deposited in the Sequence Read Archive (SRA) database (https://www.ncbi.nlm.nih.gov/sra), accession numbers: SRR22519166, SRR25519197. The original contributions presented in the study are included in the article, further inquiries can be directed to the corresponding author.
